# Gonadotropin Releasing Hormone Agonists Have an Anti-apoptotic Effect on Cumulus Cells

**DOI:** 10.3390/ijms20236045

**Published:** 2019-11-30

**Authors:** Paola Scaruffi, Sara Stigliani, Barbara Cardinali, Claudia Massarotti, Matteo Lambertini, Fausta Sozzi, Chiara Dellepiane, Domenico Franco Merlo, Paola Anserini, Lucia Del Mastro

**Affiliations:** 1 UOS Physiopathology of Human Reproduction, IRCCS Ospedale Policlinico San Martino, 16132 Genoa, Italy; paola.scaruffi@hsanmartino.it (P.S.); sara.stigliani@hsanmartino.it (S.S.); fausta.sozzi@hsanmartino.it (F.S.); paola.anserini@hsanmartino.it (P.A.); 2 Breast Unit, IRCCS Ospedale Policlinico San Martino, 16132 Genoa, Italy; barbara.cardinali@hsanmartino.it (B.C.); chiara.delle@hotmail.it (C.D.); lucia.delmastro@hsanmartino.it (L.D.M.); 3 Department of Neurosciences, Rehabilitation, Ophthalmology, Genetics, Maternal and Child Health, University of Genoa, 16132 Genoa, Italy; claudia.massarotti@gmail.com; 4 Department of Internal Medicine, University of Genoa, 16132 Genoa, Italy; 5 U.O.C. Clinica di Oncologia Medica, IRCCS Ospedale Policlinico San Martino, 16132 Genoa, Italy; 6 Infrastruttura Ricerca e Statistica, Azienda Unità Sanitaria Locale—IRCCS di Reggio Emilia, 42123 Reggio Emilia, Italy; domenico.merlo@ausl.re.it

**Keywords:** cyclophosphamide, GnRH agonist, apoptosis, oocytes, human cumulus cell-oocyte complexes

## Abstract

Background: Ovaries are sensitive to chemotherapy, which may lead to early depletion of primordial follicle reserve. One strategy for gonadal function preservation is temporary ovarian suppression with Gonadotropin Releasing Hormone agonists (GnRHa) during chemotherapy. To date, GnRHa protective mechanism of action remains not fully elucidated. Methods: We collected 260 immature cumulus cell-oocyte complexes (COC) from 111 women < 38 years old, with a normal ovarian reserve. The COC were randomly assigned to the following groups: (a) control; culture with the addition of (b) GnRHa; (c) cyclophosphamide; (d) cyclophosphamide plus GnRHa. After in vitro treatments, RNA and proteins were extracted from oocytes and cumulus cells (CC), separately. Potential effects of drugs were evaluated on GnRH receptors, apoptosis pathways, ceramide pathway, and glutathione synthesis by quantitative PCR and, whenever possible, by Western blot. Results: Cyclophosphamide triggered activation of the extrinsic pathway of apoptosis mediated by BAX in CC. The co-administration of GnRHa inhibited the apoptosis pathway in CC. According to our model, the GnRHa does not directly act on oocytes, which do not express GnRH receptors. Moreover, glutathione synthesis was decreased after GnRHa treatment both in CC and oocytes. Conclusion: Our data suggest that the protective mechanisms induced by GnRHa is mediated by an anti-apoptotic effect on CC.

## 1. Introduction

Young women with cancer or non-malignant diseases requiring treatment with cytotoxic chemotherapy are candidates for fertility preservation. The ovarian reserve is constituted by a fixed number of resting primordial follicles present at birth that are slowly depleted throughout a woman’s reproductive life [1]. The ovaries are very sensitive to cytotoxic chemotherapy agents; they can induce apoptotic death of the oocytes and the surrounding granulosa cells (GC), leading to irreversible early depletion of the primordial follicle reserve [2,3,4]. Therefore, in young females, chemotherapy can induce premature ovarian insufficiency and infertility [5].

Strategies for preservation of female gonadal function and/or fertility include temporary ovarian suppression with Gonadotropin Releasing Hormone agonists (GnRHa), oocyte/embryo or ovarian tissue cryopreservation [6,7]. In comparison with cryopreservation strategies, temporary ovarian suppression with GnRHa during chemotherapy has been developed specifically, as a technique to protect the ovaries from the gonadotoxic effect of cytotoxic systemic therapy. In addition, the main advantages are that it is simple to administer, less invasive and expensive, and does not require delay in initiating anticancer therapies. Important novel research efforts in the field have elucidated the efficacy and safety of this option, and it is now endorsed for clinical use by several guidelines [6,7,8,9]. Nevertheless, the performance of this strategy remains still debated [10]. Firstly, while consistent data proved the efficacy of the use of temporary ovarian suppression with GnRHa during chemotherapy in breast cancer patients [11], negative results were obtained in trials conducted in women with tumors other than breast cancer (i.e., hematological malignancies) [12,13]. Secondly, in terms of fertility preservation potential of GnRHa use during chemotherapy, available data are limited. The most recent meta-analyses have shown a significantly higher post-treatment pregnancy rate for premenopausal breast cancer patients treated with GnRHa during chemotherapy, as compared to those treated with cytotoxic therapy alone [8,9,11,13,14,15,16], without benefit in lymphoma patients [12,13]. Thirdly, concerning experimental preclinical data, the potential mechanisms of action for the protective effects of GnRHa during chemotherapy remains not fully clarified. Several experimental studies have been conducted in both female mice and rats. The majority of the studies showed a positive effect for GnRHa treatment in preventing chemotherapy-induced depletion of primordial follicles [17,18,19,20,21,22,23,24,25]. However, other studies did not confirm these findings [26,27,28,29]. Importantly, very limited experimental preclinical data are available in female primates and human models [30,31,32].

In regard to the activated pathway, stimulation of mouse granulosa cells with GnRHa did not induce a detectable rise in cAMP levels [27,33]. However, a recent study reported increased cAMP levels in human cortical pieces and in granulosa cell lines following GnRHa stimulation [32], thus suggesting potential different actions of GnRHa in rodents and humans. Therefore, further investigation in human models are needed because of crucial importance considering the already available clinical use for this strategy.

Hereby, we studied immature cell-oocyte complexes (COC) discarded from in vitro fertilization cycles in healthy women to investigate whether GnRHa was able to protect the oocytes from cyclophosphamide toxicity. Although chemotherapy damages primordial follicle oocytes, this was the only strategy available to us for treatment of ex vivo human COC, the major anatomic and functional unit of the ovary. The COC were cultured without the addition of drugs, in the presence of cyclophosphamide alone or GnRHa alone or both drugs. Effects of these treatments were evaluated on GnRH receptors, apoptosis pathways, ceramide pathway, and glutathione synthesis. Data were collected on both oocytes and cumulus cells (CC) by gene expression analysis.

## 2. Results

### 2.1. GnRH Receptors

In CC, only the *GnRHR-I* gene was detectable and its expression level was not modulated by treatment. In the oocytes, neither *GnRHR-I* nor *GnRHR-II* genes were expressed or induced by chemotherapy and GnRHa.

### 2.2. Apoptosis Pathway

**Figure 1:**
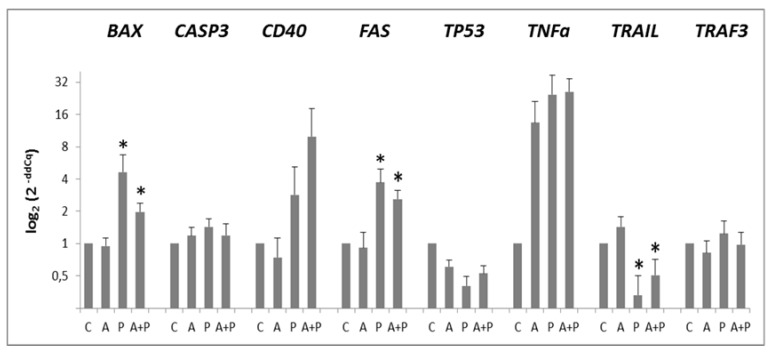
Expression levels of *BAX, CASP3, CD40, FAS, TP53, TNFα, TRAIL, TRAF3* pro-apoptotic genes in CC of cell-oocyte complexes (COC) cultured without the addition of drugs (control, C), treated with GnRHa (A), phosphoramide mustard (P), and GnRHa+phosphoramide mustard (A+P). Results are expressed as log_2_(2^-ddCq^) values using *HPRT1* as reference gene and C group as calibrator. Error bars denote standard error. *: *p* value < 0.05 in P and A+P versus A group.

In CC, *BAK1, BCL2L10, FASL*, and *TP63* pro-apoptotic genes were not expressed. Figure 1 shows expression profiles of the expressed pro-apoptotic genes in CC.

**Figure 2:**
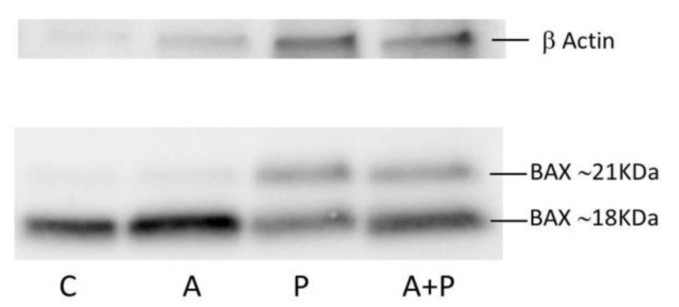
Western blot analysis of BAX in CC of COC cultured without the addition of drugs (control group, C), treated with GnRHa (A), phosphoramide mustard (P), and GnRHa+phosphoramide mustard (A+P). The rabbit polyclonal anti-BAX antibody was diluted 1:1000.

The expression of *TP53* was not modified by any treatment. Expression levels of *BAX, CASP3, FAS*, and *TRAF3* were increased in P group as compared to C group and they were diminished after GnRHa treatment. Such a behavior was particularly evident for *BAX* and *FAS* genes, that, compared to A group, were significantly up-regulated both in P (*p* = 0.0152 and *p* = 0.0006, respectively) and in A+P group (*p* = 0.0015 and *p* = 0.0006, respectively). A trend of decreasing expression of *BAX* and *FAS* was observed in A+P group than in the P one. Western Blotting analysis of BAX showed the presence of BAX~21KDa (putatively BAXα, the most common form of BAX [34]) and BAX~18KDa that could be the BAX cleaved product (Figure 2).

The expression level of BAX~21 KDa seems to increase in P compared to C and A groups, and to diminish in A+P group with respect to P group. Moreover, the ratio between BAX~21 KDa and BAX~18 KDa tends to increase following chemotherapy treatment, suggesting that cyclophosphamide may promote the synthesis of BAX~21 KDa. Due to the low amount of material and the difference in protein loading because of interfering in quantification due to Human Serum Albumin (HSA) in IVF medium, these results should only be interpreted qualitatively.

A trend for increased expression of *CD40* and *TNFα* was observed moving from A to P and A+P treatments. A statistically significant reduction of TRAIL expression was observed in the P and A+P group, as compared to A group (*p* = 0.0005 and *p* = 0.0015, respectively).

**Figure 3:**
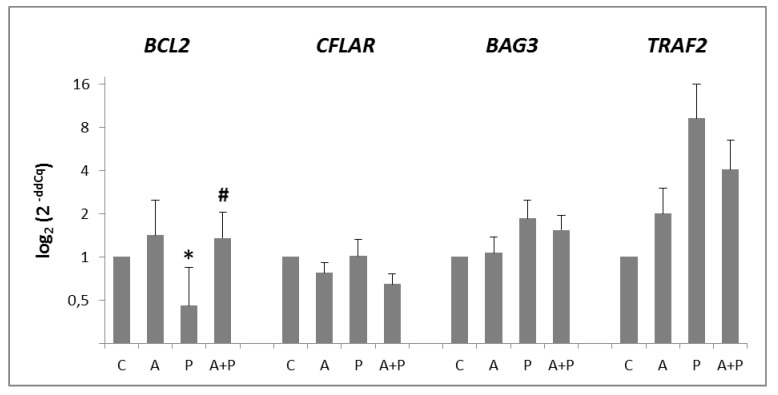
Expression levels of *BCL2, CFLAR, BAG3,* and *TRAF2* anti-apoptotic genes in CC of COC cultured without the addition of drugs (control, C), treated with GnRHa (A), phosphoramide mustard (P), and GnRHa+phosphoramide mustard (A+P). Results are expressed as log_2_ (2^-ddCq^) values using *HPRT1,* as a reference gene and the C group as a calibrator. Error bars denote standard error. *: *p* value < 0.05 in A group versus P group; #: *p* value < 0.05 in P group versus A+P group.

In CC, all anti-apoptotic genes were expressed, with the exception of *BIRC5* (Figure 3). Expression of *BCL2* gene was significantly modified by the different treatments: its expression was reduced in P group (*p* = 0.0256) and increased in A+P group (*p* = 0.0087). On the other hand, *BAG3* and *TRAF2* expression was increased in P group and slightly diminished in A+P group, without reaching statistical significance. Expression of *CFLAR* was not modified by any treatment.

In oocytes, *BAK1, CD40, FASL, TNFα*, and *TRAIL* pro-apoptotic genes were not expressed. The levels of *BAX* and *BCL2L10* mRNAs were not modified by any treatment. The expression of *CASP3, TP53, TP63,* and *TRAF3* was heterogeneously modulated by all the administered drugs (Supplementary Figure S1A). *FAS* gene was not evaluable because of its high variability among the replicates. All anti-apoptotic genes were expressed in the oocytes with the exception of *BCL2* that was detected only in the C group. The expression of *BIRC5, CFLAR, BAG3,* and *TRAF2* was not significantly modulated by treatments (Supplementary Figure S1B).

### 2.3. Sphingomyelin Pathway

The CC expressed all sphingomyelin pathway genes, whereas in the oocytes, *SPHK1* was not detected. The changes in genes of ceramide-sphingosine pathway were not statistically significant (Supplementary Figure S2), which unfortunately indicates inadequate quantity of the investigated material.

### 2.4. Glutathione-Mediated Pathway

**Figure 4:**
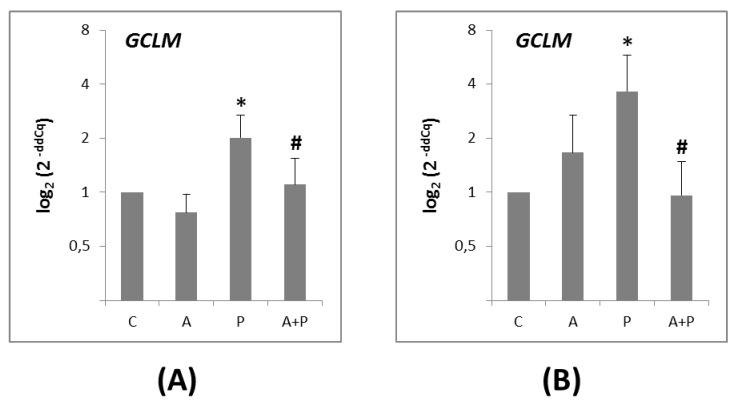
(**A**) Expression levels of *GCLM* gene in CC of COC cultured without the addition of drugs (control, C), treated with GnRHa (A), phosphoramide mustard (P), and GnRHa+phosphoramide mustard (A+P); (**B**) Expression levels of *GCLM* gene in oocytes of COC cultured without the addition of drugs (control, C), treated with GnRHa (A), phosphoramide mustard (P), and GnRHa+phosphoramide mustard (A+P). Results are expressed as log_2_ (2^-ddCq^) values using *HPRT1* as reference gene and C group as calibrator. Error bars denote standard error. *: *p* value < 0.05 in A group versus P group; #: *p* value < 0.05 in P group versus AP group.

The *GCLM* gene was expressed in both the CC (Figure 4A) and the oocytes (Figure 4B). Its expression was significantly higher in the P group, as compared to the A group in CC and oocytes (*p* = 0.0191 and *p* = 0.0421, respectively). In the A+P group, a significant reduction of *GCLM* level was observed in both CC (*p* = 0.0191) and oocytes (*p* = 0.0171) compared to the P group.

## 3. Discussion

To date, the potential protective mechanism of action of temporary ovarian suppression with GnRHa during chemotherapy is highly debated and remains not clearly elucidated. Potential hypothesized mechanisms include [10,35,36,37]: (i) interruption of Follicle-Stimulating Hormone secretion determining a hypo-gonadotropic milieu, in which follicular recruitment is inhibited and fewer primordial follicles attain the chemotherapy-sensitive stages of proliferation and follicle maturation; (ii) decrease in utero-ovarian perfusion due to the hypo-estrogenic state generated by pituitary-gonadal desensitization; (iii) activation of GnRH receptors; (iv) up-regulation of intra-gonadal anti-apoptotic molecules; or (v) protection of undifferentiated germ line stem cells. However, so far, none of these hypotheses has been definitively demonstrated. With the exception of three reports (one using ex-vivo and in vitro human cells [32] and two a mouse model [26,27]), the majority of in vivo [19,20,24,29,30,38,39,40,41,42] and in vitro [29,31,43] studies showed that GnRHa maintains primordial follicles and inhibit chemotherapy-induced apoptosis. So far, only three reports on the molecular mechanisms of function of GnRHa on cells/tissue of human origin (namely, ovarian cancer cells [43], granulosa GC [31], ex vivo and in vitro models of ovary and GC [32]) are available.

The novelty of our work lies in studying human, ex vivo COC. Indeed, we treated the COC and then analyzed the effect of GnRHa and cyclophosphamide in the oocyte and CC compartments, separately. Such an experimental set-up allowed us to closely simulate the physiological microenvironment, since COC is a fundamental anatomic and functional unit of the ovarian tissue, and oocyte growth and competence are dependent on bidirectional communication between the oocyte and the CC [44,45]. We adopted a culture system on the bases of experience from IVF. First, we used a bicarbonate buffered culture medium designed for the incubation of oocytes, supplemented with HSA, gentamicin, antioxidants, non-essential amino acids, and glucose for efficient COC survival. Second, COC incubation was performed in a controlled atmosphere with low oxygen tension (5%), in order to closely mimic conditions of the natural female environment since in the human uterus, the oxygen tension ranges between 2–8% [46]. Such a hypoxic culture reduces the generation of reactive oxygen species and related damage to oocytes [47].

Our experiments on COC showed that cyclophosphamide was mainly detrimental to the CC compartment. In fact, chemotherapy started a series of molecular signals to trigger apoptosis through the extrinsic pathway activation [48]. Specifically, in CC, the cyclophosphamide-mediated death signal seemed to be transmitted from the cell surface to the intracellular signaling effectors mainly by involvement of *FAS*, and marginally by *CD40, TRAF3* and *TNFα*, all members of the tumor necrosis factor receptor gene superfamily [49]. The CC commitment to apoptosis was mediated by the anti-apoptotic *BCL2* and the pro-apoptotic *BAX* genes [50], whose expression was down- and up-regulated after chemotherapy, respectively. At the protein level, we observed the presence of BAX~18KDa that may reflect a cellular stress due to the extended culture. Intriguingly, the trend for increased BAX~21 KDa to BAX~18 KDa ratio observed following chemotherapy suggests that cyclophosphamide may promote synthesis of BAX~21KDa that in turn, may trigger the activation of apoptosis. This effect seems to be partially counteracted by GnRHa.

Finally, the trend of *CASP3* up-regulation in CC treated with cyclophosphamide suggested that caspase-3 may be one of the “executioner” caspases activated at the end of the process. The resulting cell death was displayed by the morphological changes observed in CC in response to cyclophosphamide.

The administration of GnRHa before cyclophosphamide tended to down-regulate expression of pro-apoptotic genes as *FAS, TRAF3, BAX, CASP3*, and up-regulate transcription of anti-apoptotic *BCL2* in CC. Our molecular data did not demonstrate a direct GnRHa-mediated protection against the cytotoxic effects of cyclophosphamide in the oocytes. In fact, we observed a morphological rescue of the oocytes without any significant modulation of the analyzed genes when chemotherapy was combined with GnRHa. Taken together, these results suggest that the co-administration of GnRHa with chemotherapy inhibited the extrinsic apoptotic signaling pathway in CC only, whereas it did not impact on the oocytes. In other words, oocytes may survive the detrimental effect of cyclophosphamide thanks to the protective role of the surrounding GC. Moreover, we demonstrated at molecular level that GnRHa directly targets the CC, since the *GnRHR-I* gene was detectable only in these cells without any evident modulation by treatments. This finding in our model is in agreement with a previous report showing that GnRH receptors are mainly expressed by proliferating and luteinized GC of the growing follicles and corpus luteum, respectively [51].

Finally, the investigation of the first rate-limiting enzyme of glutathione synthesis [52] showed that in both CC and oocytes, glutathione synthesis was induced by cyclophosphamide. This is in line with prior evidence suggesting that glutathione is an important intracellular antioxidant playing several vital roles in the cells such as anti-oxidation and detoxification of xenobiotics [53]. Specifically, in the metabolism-dependent toxicity of chemotherapy, the glutathione has a role in binding phosphoramide mustard and acrolein, two toxic and highly reactive metabolites. The resulting sulfhydryl-containing compounds protect against toxicity of these alkylating agents and at the same time do not interfere with the chemotherapeutic activity of cyclophosphamide [54]. Consistently with the protective effect of GnRHa, and thus the reduced need of a detoxifying system, we observed a decrease of *GCLM* in CC and in oocytes after GnRHa treatment.

Although the expression of investigated targets was detected at low levels because of paucity of RNAs and proteins due to the nature of our COC model, we hypothesize that GnRHa directly may act on CC to protect the oocytes from cyclophosphamide toxicity by an anti-apoptotic effect. It is noteworthy that the protective role of GnRHa against cyclophosphamide has been recently excluded in ex vivo and in vitro models of human ovary and GC [32]. We believe that the opposite conclusions may be due both to the different timing of drugs administration and to the different dose of GnRHa used for experiments. In fact, in Bildik’s model, GnRHa was administered to cells at the same time as chemotherapy or 1–2 h prior to cyclophosphamide, whereas in A+P group of our model the incubation with GnRHa was performed 24 h before the addiction of chemotherapy to COC. Such a GnRHa schedule better mimics the protocol used in the clinic with patients starting GnRHa at least 1 week before chemotherapy initiation [55]. Moreover, in Bildik’s experiments the GnRHa was used at 10-20-40 nM, that is two order of magnitude lower than ours (1 µM). Thus, we suggest that prior and longer exposure to a higher dose of GnRHa facilitates in CC, and indirectly in the oocytes, the activation of those molecular pathways that lead to decrease cell damage and follicular apoptosis during chemotherapy.

Our data suggest that the protective gonadal effect of GnRHa treatment during chemotherapy with cyclophosphamide may be indirect and mediated through the surrounding CC. Despite this being a preliminary study, it adds knowledge on the potential mechanisms of action for the protective effects of GnRHa during chemotherapy, a therapeutic strategy that is now endorsed for clinical use by several guidelines. In order to overcome the intrinsic limitation of the presented study in terms of transcript/proteome coverage, future wide analyses could complement our results by larger depth investigations of the precise molecular mechanisms [56,57].

## 4. Materials and Methods

### 4.1. COC Collection

Human immature COC, retrieved for assisted reproduction and discarded because they were not suitable for in vitro fertilization, were collected from 111 women between March 2014 to March 2017 by transvaginal oocyte aspiration 36 h after injection of human chorionic gonadotropin. All COC derived from women < 38 years old (range: 22–38 years), with a normal ovarian reserve [58], without any ovarian pathology. A standard controlled ovarian stimulation protocol including gonadotropin administration in combination with GnRH antagonist was applied as previously described [59]. Under SZX7 stereomicroscope (Olympus Corporation, Tokyo, Japan), evaluation of COC maturity was based upon the expansion and radiance of the cumulus-corona complex, which surrounds the oocyte [60]. Absence of expanded cumulus-corona complex was associated with oocyte immaturity. Immature COC were incubated in Sydney IVF Fertilization medium (Cook Medical, Bloomington, IN, USA) at 37 °C in a humidified atmosphere of 6% CO_2_, 5% O_2_ using Galaxy 48R incubator (New Brunswick Scientific, Edison, NJ, USA). COC culture was performed in Nunc™ 4-well IVF dishes (Thermo Fisher Scientific, Waltham, MA, USA).

### 4.2. Ethical Approval

Human immature COC, not suitable for IVF treatment, were donated after written informed consent. The study was approved by the Ethics Committee of Regione Liguria (approval n. 032REG2013, approved on November 12th, 2013).

### 4.3. Study Design

We treated whole COC and then analyzed the effect of GnRHa and cyclophosphamide in the oocyte and CC compartments, separately. The COC were randomly assigned to each treatment group using a computer-generated randomization list: (i) Control group (no drugs) (C); (ii) GnRHa-treated group (A); (iii) Chemotherapy-treated group (P); (iv) Chemotherapy plus GnRHa-treated group (A+P). A total of 260 COC were analyzed, we utilized 200 COC for gene expression analysis (five replicates of the four experimental groups, pools of 10 oocytes and relative CC), and 60 COC for protein analysis (three replicates of the four experimental groups, pools of 5 oocytes and relative CC).

### 4.4. In Vitro Treatments

The control group was incubated for 48 h in the Sydney IVF Fertilization medium. At t0 in group A the GnRHa (1 µM triptorelin, the concentration that obtain the maximum protection effect according to Imai et al. [31]; Sigma-Aldrich S.r.l., Milan, Italy) was added, and incubation was performed for 48 h. At t0 + 24 h in group P the chemotherapy (30 µM) was added and incubation was performed for 24 h. We chose the chemotherapy dose by performing preliminary MTT assay on cumulus cells. At t0 in group A+P the GnRHa (1 µM) was added, the incubation was performed for 24 h, and then the chemotherapy (30 µM) was added for additional 24 h of incubation. Phosphamide mustard was used as a chemotherapy agent considering it is the active metabolite of cyclophosphamide (Open Chemical Repository, Developmental Therapeutics Program, National Institutes of Health, Rockville, MD, USA).

### 4.5. RNA Isolation, Quantitation and Retro-Transcription

At the end of treatment, the oocytes were denuded of CC by enzymatic treatment with 80 IU/mL hyaluronidase solution (Origio, Målov, Denmark). Oocytes and CC were separately washed in Phosphate buffered saline (PBS), frozen in Qiazol (Qiagen, Hilden, Germany), and stored at −80 °C. Pools of 10 oocytes and CC were used for molecular analyses separately. Total RNA was extracted from each pool using the miRNeasy Micro kit (Qiagen), according to the manufacturer’s procedure. Quantification and quality control of total RNA were performed by RNA 6000 Pico kit on a 2100 Bioanalyzer (Agilent Technologies, Santa Clara, CA, USA). The RNA (2 ng or 10 ng for oocytes and CC, respectively) was amplified and reverse-transcribed using Ovation Pico WTA System V2 (NuGEN, AC Leek, The Netherlands) following the manufacturer’s instructions. cDNA was purified by Mini Elute kit (Qiagen) and diluted 1:10 for molecular analyses.

### 4.6. Quantitative PCR (qPCR)

We investigated the following targets: (i) GnRH receptor genes (*GnRHR-I* and *GnRHR-II*); (ii) Pro-apoptotic (*BAX, BAK, BCL2L10, CD40, TRAF3, TP53, TP63, CASP3, TNF-α, FAS, FASL,* and *TRAIL*) and anti-apoptotic (*BCL2, BIRC5, CFLAR, BAG3,* and *TRAF2*) genes; (iii) Sphingomyelin pathway: sphingomyelin phosphodiesterase 1 (*SMPD1*), ceramide synthase 1 (*CERS1*), *N*-acylsphingosine amidohydrolase 1 (*ASAH1*), and sphingosine-1-kinase (*SPHK1*) genes; (iv) glutamate-cysteine ligase (*GCLM*), the first rate limiting enzyme of glutathione synthesis. *HPRT1* was used as reference gene. Specific TaqMan^®^ assays were performed in triplicate in a Mastercycler epRealPlex2S system (Eppendorf, Hamburg, Germany). Negative control (water as template) was run simultaneously.

### 4.7. Western Blot Analysis

The CC were lysated in RIPA Buffer with 0.1 mM Phenylmethanesulfonyl fluoride (PMSF, Cell Signaling Technology Danvers, MA, USA) and protease inhibitors (Sigma S.r.l.). The lysates were stored at −20 °C. Protein amount was evaluated using Pierce™ 660 nm Protein Assay Reagent (Pierce Biotechnology, Rockford, IL, USA) and micro DC Protein Assay (Biorad Laboratories, Hercules, CA, USA). Proteins were resolved in 4–20% SDS Mini Protean TGX Precast gels (Biorad) and transferred on a PVDF membrane using a Trans-Blot Turbo Trasfert System (Biorad). Membranes were blocked with 1% no fat milk in PBS containing 0.05% Tween 20 for 1 h and probed with rabbit polyclonal anti-BAX (Cell Signaling Technology) antibody overnight at +4 °C. Bound antibody was detected using horseradish peroxidase (HRP)-conjugated anti-rabbit secondary antibody (Cell Signaling Technology) incubated 1 h at room temperature. Proteins were detected by chemiluminescence (Clarity Western ECL Substrate, Biorad) using UVITEC Imaging System (Cleaver Scientific, Cambridge, UK). Quantification was performed after normalization on the β-actin band immunoassayed (HRP-conjugated β-Actin Rabbit monoclonal antibody, Cell Signaling Technology).

### 4.8. Statistical Analysis

RealPlex software v.2.0 (Eppendorf) was used to determine quantification cycle (Cq). Results of qPCR were expressed as relative values (ddCq method [61] using *HPRT1* as reference gene [62]) and control group as calibrator). Mann-Whitney test (MedCalc^®^ software, Mariakerke, Belgium) was used to test the significance of the difference between the sum of the ranks of each target gene measured in each treatment groups. Between treatment groups differences were considered statistically significant at a two-sided *p*-value < 0.05.

## Supplementary Materials

Supplementary Materials can be found at https://www.mdpi.com/1422-0067/20/23/6045/s1.

## Abbreviations

GnRHa	Gonadotropin Releasing Hormone agonists
COC	Cumulus cell-Oocyte Complexes
CC	Cumulus Cells
GC	Granulosa Cells
IVF	In Vitro Fertilization
HSA	Human Serum Albumin
