# Supplementary material for: Gonadotropin Releasing Hormone Agonists Have an Anti-apoptotic Effect on Cumulus Cells
Source: Int J Mol Sci. 2019 Nov 30;20(23):6045. doi: 10.3390/ijms20236045 (PMC6928931; doi:10.3390/ijms20236045)
Supplement: Supplementary file 1 [file ijms-20-06045-s001.pdf]

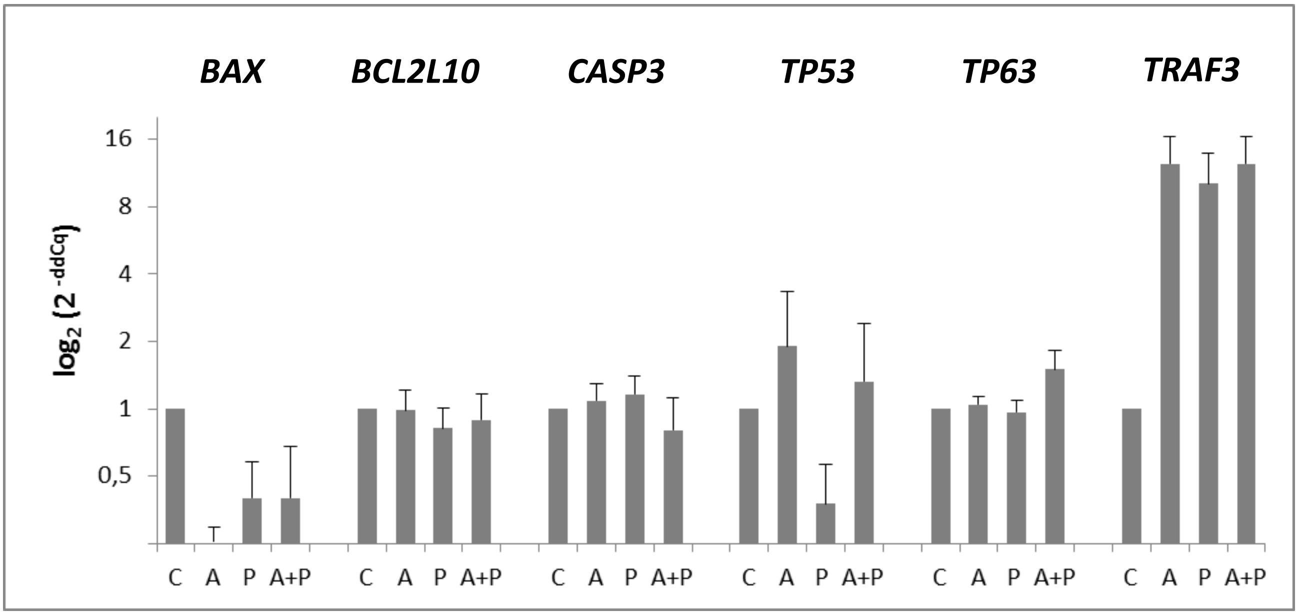

(A)

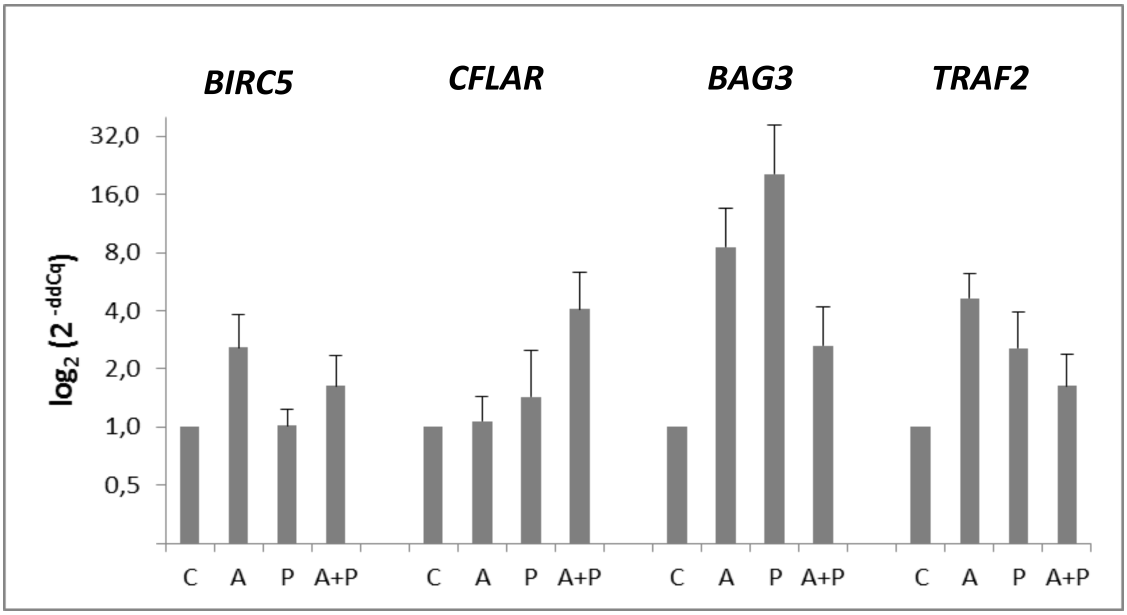

(B)

Supplementary Figure 1: (**A**) Expression levels of *BAX, BCL2L10, CASP3, TP53, TP63, TRAF3* apoptotic genes in oocytes of COC cultured without the addition of drugs (control, C), treated with GnRHa (A), phosphoramide mustard (P), and GnRHa+phosphoramide mustard (A+P); (**B**) Expression levels of *BIRC5, CFLAR, BAG3, TRAF2* anti-apoptotic genes in oocytes of COC cultured without the addition of drugs (control, C), treated with GnRHa (A), phosphoramide mustard (P), and GnRHa+phosphoramide mustard (A+P). Results are expressed as $\log_2(2^{-ddCq})$ values using *HPRT1* as reference gene and C group as calibrator. Error bars denote standard error.

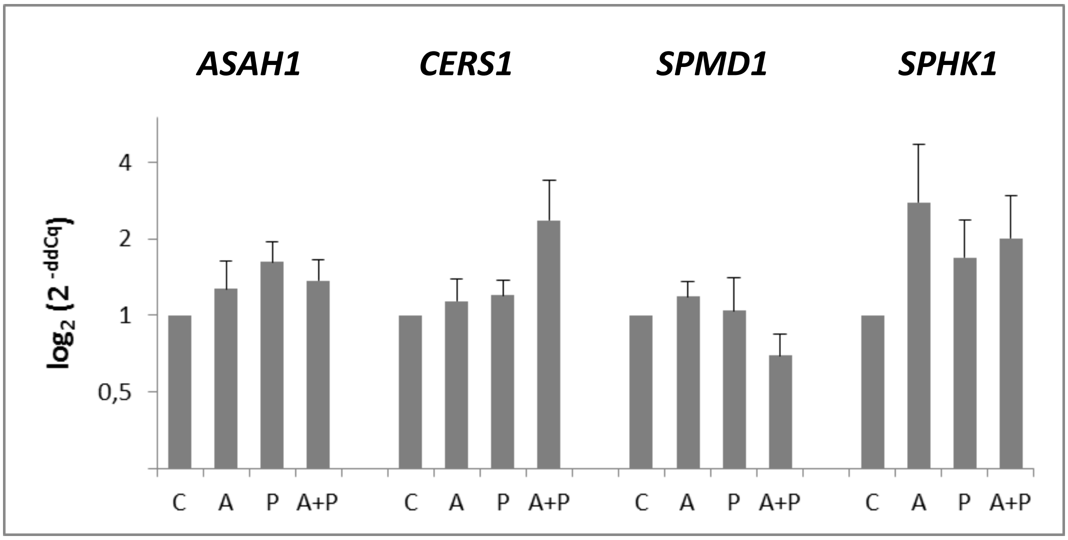

(A)

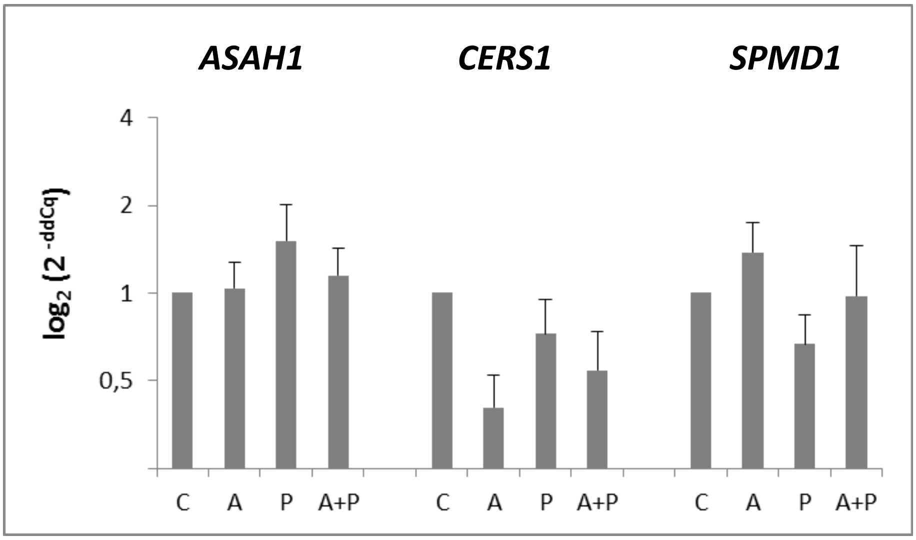

(B)

Supplementary Figure 2: (**A**) Expression levels of sphingomyelin pathway genes in CC of COC cultured without the addition of drugs (control, C), treated with GnRHa (A), phosphoramide mustard (P), and GnRHa+phosphoramide mustard (A+P); (**B**) Expression levels of sphingomyelin pathway genes in oocytes of COC cultured without the addition of drugs (control, C), treated with GnRHa (A), phosphoramide mustard (P), and GnRHa+phosphoramide mustard (A+P). Results are expressed as $\log_2(2^{-ddCq})$ values using *HPRT1* as reference gene and C group as calibrator. Error bars denote standard error.
